# Mitochondrial niches of residual disease in EGFR-mutant NSCLC: immune-constrained persistence and therapeutic interception

**DOI:** 10.3389/fimmu.2026.1908826

**Published:** 2026-08-24

**Authors:** Zhanhao Liang, Xutong Zhao, Mingru Jiang, Meizhu Zhou, Miao Cheng, Shibiao Liu, Yi Zhao

**Affiliations:** 1 First Affiliated Hospital, Dalian Medical University, Dalian, Liaoning, China; 2 College of Health-Preservation and Wellness, Dalian Medical University, Dalian, Liaoning, China; 3 Department of Vascular Surgery, Beijing Tsinghua Changgung Hospital, School of Clinical Medicine, Tsinghua Medicine, Tsinghua University, Beijing, China; 4 Department of Oncology, The First Affiliated Hospital of Dalian Medical University, Dalian, Liaoning, China

**Keywords:** EGFR-mutant non-small cell lung cancer, drug-tolerant persisters, minimal residual disease, mitochondrial adaptation, tumor microenvironment

## Abstract

Therapeutic failure in EGFR-mutant non-small cell lung cancer (NSCLC) is commonly viewed as a consequence of acquired resistance, but this perspective may overlook an earlier and potentially more tractable phase marked by the survival of residual cells after effective EGFR inhibition. In this Review, we define the mitochondrial niche as a temporally bounded, hypothesis-generating model of early residual disease in which tumor-cell mitochondrial stress adaptation is considered alongside fibroblast–matrix support and incomplete immune clearance. The conceptual contribution of this framework is to place independently studied tumor-intrinsic, stromal, and immune processes on a common temporal axis and to generate testable questions about their potential interaction; whether these processes coexist and function as a coordinated niche in EGFR-mutant residual disease has not yet been directly demonstrated. We distinguish DTPs and cycling persisters as experimentally defined cell states, MRD as a clinical detection state, and acquired resistance as overt regrowth under continued therapy. In selected preclinical models, EGFR-targeted therapy is associated with survival states involving mitochondrial fuel use, redox control, organelle-quality surveillance, or apoptotic restraint. In parallel, selected cancer-associated fibroblast states may buffer therapy-induced stress, whereas treatment-associated inflammatory remodeling often fails to generate productive antitumor immunity. By treating residual disease as a distinct, low-burden and testable state rather than as direct evidence of a single persister ecology, this framework prioritizes candidate biomarker classes and mechanism-based interventions for prospective validation before stable resistance is established. Candidate readouts include driver-ctDNA kinetics, residual-lesion status, stromal-state markers, and selected immune features, all requiring prospective validation.

## 1 Introduction

EGFR-mutant non-small cell lung cancer (NSCLC) has become one of the clearest demonstrations that precise oncogene targeting can alter the natural history of a solid tumor. Yet, even in the era of osimertinib, durable eradication remains uncommon. The clinical success of EGFR blockade has therefore sharpened, rather than resolved, a more fundamental biological question: why do a small fraction of cancer cells survive despite profound target inhibition and later seed relapse? Emerging MRD data have sharpened this question by showing that molecular recurrence can precede radiographic progression. However, ctDNA-defined MRD does not by itself specify the underlying cellular state of that residual disease (1, 2). Traditional resistance taxonomies remain essential for explaining overt progression, including secondary EGFR mutations, bypass signaling, and lineage plasticity. The question addressed in this Review is not whether these mechanisms matter, but how residual cells remain viable before one mechanism becomes dominant, stable, or clinically detectable. The mitochondrial-niche framework is therefore used here as a complementary organizing lens for the residual-disease interval, rather than as an alternative to existing multi-pathway resistance models. Earlier, largely preclinical microenvironment studies suggested that survival under EGFR inhibition is not purely cell-autonomous: stromal fibroblasts and matrix-rich conditions can buffer EGFR-TKI effects, and collagen I can maintain P70S6K/mTOR signaling despite suppression of EGFR, ERK, and Akt outputs (3). Because the collagen I study was conducted mainly in early-generation EGFR-TKI contexts, including gefitinib-based experimental models, we use it as proof-of-principle for matrix-mediated buffering rather than as direct evidence that the same mechanism operates under first-line osimertinib pharmacology. These observations suggest that, in some settings, an early barrier to cure may involve not only genomic evolution but also a permissive microenvironmental niche that buffers the initial drug insult. What has changed in recent years is that residual-disease biology has become more mechanistically tractable. Residual disease is increasingly being linked to potentially actionable stress-adaptation programs rather than only to a generic notion of “stromal protection.” In patient-derived models, immunocompetent systems, and clinical specimens obtained on targeted therapy, a focal adhesion kinase (FAK)-YAP signaling axis has been shown to support drug-tolerant persister (DTP) cells and residual disease during oncogene-directed treatment (4). At the same time, adaptive resistance has been tied to mitochondrial maintenance: NCOA4-mediated ferritinophagy preserves iron handling and oxidative metabolism under osimertinib pressure (5), whereas a DPP4-CPT1A axis promotes fatty acid uptake, fatty acid oxidation, and oxidative phosphorylation in persister cells (6). Taken together, these studies suggest that, in preclinical models, a subset of EGFR-mutant cells engages active stress-adaptation programs that include mitochondrial maintenance, rather than being explained solely by passive quiescence. Importantly, available preclinical data suggest that some mitochondrial stress-adaptation states may not be sustained by tumor cells alone. A recent preclinical study identified an RGS5^+^MYL9^+^ CAF population associated with EGFR-TKI resistance and poor prognosis; in that system, these CAFs were recruited to DTP niches through CCL11 signaling and formed Miro1/RhoA-dependent tunneling nanotubes that accepted damaged mitochondria from osimertinib-treated tumor cells (7). This study provides a mechanistically plausible example of stromal organelle-stress handling during EGFR-TKI tolerance, but the frequency, spatial distribution, and quantitative contribution of mitochondrial transfer across diverse patient tumors remain unresolved. Accordingly, mitochondrial transfer should be presented as a context-dependent stromal mechanism that may sustain selected DTP niches, not as a general explanation for residual-disease persistence. The residual niche is also immunologically consequential. EGFR-mutant NSCLC is frequently characterized by low cytotoxic T-cell infiltration, reduced T-cell clonality, and enrichment of the CD73/adenosine pathway, indicating that persistence unfolds within a microenvironment already biased toward immune inefficiency (8). In parallel, the persister state is beginning to yield therapeutically exploitable surface programs: TROP2 is enriched in EGFR-TKI-exposed residual disease, and TROP2-directed CAR-T cells prolonged relapse-free survival and eliminated osimertinib-induced DTP-like disease in selected xenograft and patient-derived xenograft models (9). Because these *in vivo* studies were performed in immunodeficient models, including NSG mice, they support targetability of TROP2-positive persister-like cells but do not fully model endogenous antitumor immunity, CAR-T trafficking, on-target/off-tumor toxicity, or clinical durability in patients with EGFR-mutant NSCLC. Collectively, these studies independently implicate mitochondrial adaptation, selected CAF and matrix states, and incomplete immune clearance in EGFR-TKI persistence. They do not yet establish that these processes coexist or interact within the same residual lesion. We therefore use the term “mitochondrial niche” as a temporally bounded, hypothesis-generating model that links experimentally defined persister states to the clinically detectable residual-disease interval without assuming that all proposed components coexist in every tumor. Its added value is operational: it distinguishes DTPs, cycling persisters, MRD, and acquired resistance; matches each state to the evidence systems capable of resolving it; and prioritizes biomarkers and time-windowed interventions before stable resistance emerges.

## 2 Literature identification, study selection, and evidence weighting

This Review is a structured narrative synthesis of clinical, translational, and mechanistic evidence relevant to residual disease in EGFR-mutant NSCLC. A systematic-style literature search was performed in PubMed/MEDLINE, Embase, and Web of Science Core Collection from database inception to 31 January 2026, with the final search updated on 31 January 2026. Additional relevant studies were identified through manual screening of reference lists from key primary articles and recent high-quality reviews. Search terms combined disease-specific concepts with residual-disease, metabolic, stromal, and immune terms, including but not limited to: “EGFR-mutant NSCLC,” “EGFR-mutated lung adenocarcinoma,” “drug-tolerant persisters,” “persisters,” “minimal residual disease,” “molecular residual disease,” “ctDNA,” “mitochondria,” “oxidative phosphorylation,” “mitophagy,” “ferritinophagy,” “ferroptosis,” “fatty acid oxidation,” “cancer-associated fibroblasts,” “CAF,” “extracellular matrix,” and “immune microenvironment.” Database-specific controlled vocabulary and free-text terms were combined using Boolean operators. Representative search strings are provided in the **Supplementary Methods**. Studies were considered eligible when they were published in English and were directly relevant to EGFR-mutant NSCLC residual disease, drug-tolerant persistence, MRD, mitochondrial adaptation, stromal buffering, or immune-microenvironment remodeling under EGFR-targeted therapy. Clinical longitudinal studies, paired patient-sample analyses, patient-derived translational models, and mechanistic experimental studies were included according to their relevance to disease state, treatment timing, and mechanistic interpretability. Studies focused exclusively on established end-stage resistance mechanisms without relevance to early persistence or residual disease were used selectively as background rather than as core evidence. Cross-disease studies were retained only when they informed conserved biological processes, and were not used to define disease-state relevance in EGFR-mutant NSCLC. Screening was performed in two stages: title/abstract review followed by full-text assessment. Thematic data extraction focused on disease context, model system, treatment state, proposed mechanism, and degree of relevance to early persistence, MRD, or acquired resistance. Evidence was then weighted using a predefined interpretive framework: Level 1, longitudinal human/clinical evidence anchoring disease-state relevance and prognostic timing; Level 2, patient-derived translational evidence supporting biological plausibility in human disease context; Level 3, mechanistic preclinical evidence informing causal circuitry and therapeutic hypotheses; and supportive evidence, cross-disease studies informing conserved adaptive processes. When evidence across levels was discordant, priority was given to EGFR-mutant NSCLC clinical studies for disease-state interpretation and to mechanistic experimental systems for causal inference.

## 3 Why a “mitochondrial niche” framework is needed, rather than another review of EGFR-TKI resistance mechanisms

### 3.1 Clinical benefit has outpaced biological explanation

EGFR-mutant NSCLC is one of the few solid tumors in which genotype-directed therapy has repeatedly reshaped the clinical course of disease. In FLAURA, first-line osimertinib nearly doubled median progression-free survival relative to first-generation EGFR TKIs, and later overall-survival analyses confirmed a survival advantage. Yet these gains did not translate into routine eradication; progression remained the rule, not the exception (10). This disconnect matters conceptually. If potent inhibition of the driver oncogene were sufficient, relapse would be rare and largely reducible to a finite list of late genomic escape events. Instead, the clinical phenotype is one of deep but incomplete response, followed by persistence and eventual regrowth. That pattern implies that the central biological problem is not merely whether EGFR is inhibited, but which cells remain viable after it is inhibited and under what conditions they are maintained. Clinically, this first point is anchored most strongly by randomized phase III trial data. However, trial efficacy alone does not explain *why* residual disease survives. For that, one must move to post-progression molecular studies. In the FLAURA plasma ctDNA resistance analysis, MET amplification and EGFR C797S were recurrent candidate mechanisms after first-line osimertinib, whereas acquired EGFR T790M-mediated resistance was not observed. However, these data should be interpreted as evidence of heterogeneity among plasma-detectable genomic mechanisms, not as proof that unexplained progression necessarily reflects non-genetic persistence (11). Because ctDNA-based profiling is constrained by tumor DNA shedding, assay sensitivity, copy-number detection, tissue availability, and incomplete capture of spatial or histologic transformation, undetected mechanisms may reflect either true non-genetic adaptation or insufficient sampling of genetic, lineage, and microenvironmental resistance mechanisms. A prospective tissue-and-plasma study reached a similar conclusion in real-world practice: resistance was molecularly heterogeneous, tissue and plasma showed poor concordance for resistance mechanisms, and a substantial fraction of progression could not be fully captured by any single assay (12). Taken together, the available clinical and translational literature suggests that an endpoint catalogue of resistance mutations is unlikely to fully account for recurrence in all settings.

### 3.2 Resistance mechanisms and persister states describe complementary temporal layers of treatment failure

The classic resistance framework remains indispensable because it identifies actionable genetic, bypass, and lineage-associated alterations. Its principal limitation for the present question is temporal sampling: most resistance studies characterize disease at radiographic progression and therefore provide less direct information about the composition of the earlier residual population. This limitation does not imply that genetic evolution begins only after persistence or that all resistant tumors arise through a preceding mitochondrial state. The most important corrective to that view came from cell-state biology. Sharma and colleagues first established that a small subpopulation of cancer cells can enter a reversible drug-tolerant state rather than undergoing immediate elimination. Hata and colleagues then showed, in EGFR-mutant lung cancer, that resistant clones may either pre-exist or evolve from initially drug-tolerant cells. Ramirez and colleagues sharpened the point further: even a single persister bottleneck can generate diverse downstream resistance phenotypes. In some models, stable resistance evolves from an earlier drug-tolerant population; in others, pre-existing resistant clones or alternative cell states may contribute from the outset (13–15). A complete account of treatment failure therefore requires both endpoint resistance profiling and longitudinal analysis of the residual-disease interval. Subsequent work has shown that this early survival state is not biologically featureless. In EGFR-mutant models, EGFR inhibition can induce a senescence-like dormant state marked by high YAP/TEAD activity, and pharmacologic interference with this program increases cell killing rather than merely delaying outgrowth (16). Single-cell transcriptomic and epigenomic profiling has further demonstrated that tolerant and fully resistant states are neither identical nor linearly interchangeable; instead, tolerant populations carry distinct transcriptional programs, including CD74-dependent survival signals that can directly enable regrowth under osimertinib (17). More recently, genome-wide CRISPR loss- and gain-of-function screens converged again on Hippo-YAP/TEAD as a recurrent non-genetic survival axis in EGFR-mutant lung cancer persisters (18). Taken together, these studies support the view that persister biology reflects a non-random adaptive state with recurring, but not yet fully unified, survival programs. That is precisely why a review focused only on endpoint resistance mechanisms is now too coarse for the field.

### 3.3 Why a mitochondrial niche framework may help organize the biology of residual disease

Once persistence is recognized as a structured state, the next question becomes what sustains it. Here, metabolic adaptation may provide a useful complementary organizing principle alongside bypass signaling pathways. Long before the recent wave of ferritinophagy and FAO studies, Martin and colleagues showed that osimertinib suppresses glycolysis in sensitive EGFR-mutant cells while creating a strict dependence on mitochondrial oxidative phosphorylation; importantly, OxPhos inhibitors delayed the development of resistance (19). In parallel, Taniguchi and colleagues demonstrated that osimertinib exposure can activate AXL through feedback relief, maintaining survival and facilitating the emergence of tolerant cells. These observations are conceptually linked: both point to a therapy-induced survival state that is dynamic, non-genetic, and metabolically reconfigured rather than simply mutation-defined (20). We use the term “mitochondrial niche” to describe a proposed residual-disease model in which tumor-cell mitochondrial adaptation is considered alongside cell-state plasticity, CAF- and matrix-mediated support, and incomplete immune clearance. Evidence supports each of these components to different degrees, but their causal integration within the same EGFR-mutant residual-disease setting remains unproven. The framework does not assume universal mitochondrial dependence. Instead, it places these independently supported processes on a common temporal axis and asks which residual states are mitochondria-dependent, which extrinsic components are necessary rather than associative, and when these dependencies remain therapeutically accessible. The operational distinctions among residual-disease states, the proposed niche layers, and the evidence-stratified candidate strategies for therapeutic interception are summarized in **Tables 1**–**3**.

**Table 1:** Conceptual and operational distinctions between DTPs, cycling persisters, MRD, and acquired resistance in EGFR-mutant NSCLC. Summary of the definitions, temporal context, proliferative features, genetic stability, clinical correlates, detection methods, and usage boundaries of key residual-disease terms used in this review.

Term	Definition	Tempora I position	Proliferative?	Stable genetic alteration?	Clinical counterpart	Typical detection methods	Usage boundary in this review
DTPs	Reversible drug-surviving cells that persist under therapy without requiring fixed resistance mutations	Early on- treatment	Usually low- cycling or non- cycling	No not required	Cellular basis of early residual survival under EGFR-TKI therapy	Drug-tolerance assays lineage tracing single-cell profiling	Use as the umbrella cell-state term for early surviving cells: not synonymous with MRD or acquired resistance
Cycling persisters	A subset of persisters that continues to proliferate under continuous drug exposure	Early on- treatment	Yes	No not required	Residual cells with regrowth potential during treatment	Lineage tracing. proliferation reporters. single-cell profiling	Use only for the proliferative subset within DTPs
MRD	Residual disease below radiographic detectability usually identified by sensitive molecular assays	Residual phase	Variable / not definitional	Variable	ctDNA-positive imaging-negative residual disease	aDNA assays serial plasma monitoring sometimes CTC analysis	Use as a clinical disease-burden term. not a coll-state term
Acquired resistance	Overt relapse during therapy caused by durable adaptations that permit growth despite treatment	Progression phase	Yes	Often yes	Radiographic or clinical progression on EGFR-targeted therapy	Repeat biopsy. plasma NGS/ctDNA histologic reassessment	Use only for established progressive disease. not for early persisters or MRD

DTPs and cycling persisters are discussed as cell-state entities, MRD as a clinical residual-disease state, and acquired resistance as overt progressive disease under ongoing treatment.

**Table 2:** Evidence-informed niche layers contributing to residual disease in EGFR-mutant NSCLC.

Niche layer	Key mechanisms	Functional consequence	Therapeutic implication
Tumor-intrinsic mitochondrial adaptation	OxPhos dependence. redox buffering. mitophagy. apoptotic restraint	Enables survival under EGFR inhibition	Target mitochondrial fitness and survival pathways
Tumor cell-state plasticity	Drug-tolerant persisters. cycling persisters. adaptive rewiring	Maintains a heterogeneous residual reservoir	Intercept persister states before stable resistance emerges
CAF-mediated buffering	HGF/IGF-1/NRG1 signaling. fibroblast-state heterogeneity	Sustains tumor survival despite EGFR blockade	Combine EGFR-TKI with stromal-targeting strategies
Matrix and mechanotransduction	Integrin. FAK-YAP. ILK-SFK-YAP signaling	Reinforces persistence and residual-cell fitness	Target adhesion-dependent survival signaling
Metabolic and organelle support by the niche	Metabolite exchange. kynurenine-AhR signaling. damaged mitochondrial transfer	Buffers therapy-induced metabolic stress	Disrupt tumor-stroma metabolic and organelle crosstalk
Pretreatment immune-cold baseline	Low MHC-I. weak CD8+ infiltration. Treg-biased microenvironment	Favors early residual survival	Rationally integrate immune-remodeling approaches
Miswired inflammation and myeloid dominance	IFN/CXCL10-linked stress signaling. CD24- associated escape. M2-like TAMs	Prevents effective immune clearance of MRD	Reprogram myeloid cells and optimize treatment timing
Residual-disease surface vulnerabilities	CD24 and other persister-associated surface programs	Creates targetable liabilities in residual cells	Develop antibody- and cell-based interception strategies

Summary of the proposed niche layers relevant to early persistence and residual disease, including their representative mechanisms, biological consequences, and candidate therapeutic implications. This table presents the mitochondrial-niche framework as a structured interpretive model rather than a uniformly established mechanism.

**Table 3:** Evidence-stratified candidate strategies for therapeutic interception during the early persistence and residual-disease window in EGFR-mutant NSCLC.

Intervention strategy	Target/pathway	Action level	Best intervention timing	Main evidence level	Expected clinical value	Limitations / unresolved issues
Osimertinib + transient IGF-1R inhibition	IGF-IR/FOXA1-IGF-1R adaptive survival signaling	Tumor-intrinsic	Early persistence window before tolerant cells stabilize	Preclinical *in vitro* + xenograft evidence	May prevent establishment of a viable persister reservoir and deepen initial response	No biomarker-defined patient selection yet; durability and safety of time-limited GF-1R cotargeting remain uncertain
Osimertinib + y-secretase inhibitor	Notch / NOTCH1 signating	Tumor-intrinsic	Early DTP window	Prectinical mechanistic evidence	May suppress DTP maintenance before transition to overt resistance	Clinical tolerability of Notch blockade and the exact responsive subgroup remain unresolved
Anti-CD24 strategy with EGFR-TKI backbone	CD24 innate immune-evasion surface program	Immune-surface targeting	Residual-disease window after initial debulking when surviving cells display targetable surface remodeling	Translational preclinical evidence	May enhance macrophage. mediated clearance of persisters and residual cells	Clinical validation is still lacking optimal combination partner, sequencing and toxicity profile remain undefined
CAR-T / CAR-NK-based elimination of persisters	EGFR-directed cellular therapy	Immune-surface targeting	MRD / low-burden residual phase when target burden is low and escape diversity is still limited	Translational preclinical evidence	Direct killing of DTPCs CAR-NK may 00 especially attractive for residual disease because of activity against DTP and resistant states	No established clinical workflow in EGFR-mutant MRD: antigen heterogeneity trafficking on-target toxicity. and immune suppression remain major barriers
Sequential PD-1 + VEGFR2 blockede after EGFR-TKI priming	PD-1 / VEGFR2; CD8+ T-cell reactivation after EGFR inhibition	Immune-metabolic	After initial EGFR-TKI debulking not as simultaneous up-front triple therapy	Preclinical immunocompetent model evidence	May convert transient TKI-induced immune awakening into more durable antitumor immunity	Strong schedule dependence: human validation is limited: benefit may depend on pre-existing immune context

Summary of selected tumor-intrinsic, immune-surface, and immune-metabolic interception strategies, with emphasis on target pathway, preferred timing, evidence level, expected clinical value, and current limitations. These approaches are presented as hypothesis-generating options rather than established standards of care.

## 4 Harmonizing terminology and temporal scale: DTPs, cycling persisters, MRD, and acquired resistance are not interchangeable

### 4.1 Definitions: the field needs sharper boundaries before it needs more pathways

A recurring weakness in the EGFR-TKI literature is conceptual compression: drug-tolerant persisters (DTPs), cycling persisters, molecular residual disease (MRD), and acquired resistance are often treated as sequential synonyms, although they are better regarded as related but non-equivalent biological states. Operationally, in this Review, DTPs refer to tumor cells that remain viable under otherwise effective EGFR inhibition before a stable, clinically dominant resistance mechanism is established or detectable by standard assays. This definition does not assume that DTP-enriched populations are genetically uniform or mutation-free; rare pre-existing or emerging subclonal alterations may coexist with reversible transcriptional, epigenetic, metabolic, and microenvironmentally supported tolerance programs. In EGFR-mutant lung cancer models, this state is increasingly being resolved as a structured, but heterogeneous, adaptive phenotype rather than a nonspecific survival residue. Nie and colleagues showed that persistent tolerance under EGFR-TKI pressure is accompanied by coordinated metabolic rewiring—specifically acetylcholine accumulation, YAP-dependent induction of choline acetyltransferase, and pharmacologically manipulable tolerance *in vitro* and *in vivo*—arguing that DTP formation is an active adaptive program rather than passive underexposure to drug (21). Oren and colleagues refined this terminology in the EGFR-mutant PC9 Watermelon lineage-tracing model, showing that most persisters are non-cycling, whereas a rare subset remains proliferative under continuous osimertinib exposure; in that system, cycling and non-cycling persisters arose from distinct lineages and carried different transcriptional and metabolic programs (22). However, this dichotomy should be treated as a high-resolution experimental classification rather than as a fixed binary expected to hold across all three-dimensional, organoid, xenograft, or patient-tumor contexts. In spatially structured tumors, gradients of drug exposure, oxygen, nutrients, extracellular matrix, stromal contact, and immune pressure may generate a continuum of cell-cycle states rather than two discrete persister categories. Together, these data justify a terminological distinction: DTP is the umbrella state, whereas cycling persister denotes a biologically narrower subpopulation with retained proliferative competence under treatment. By contrast, MRD is not a cell state definition but a disease burden definition. MRD refers to residual cancer that remains below conventional radiographic detectability yet is still biologically relevant and clinically consequential. In resected EGFR-mutant NSCLC, Jung and colleagues showed that longitudinal plasma ctDNA monitoring identifies patients at high risk of recurrence, with postoperative MRD positivity independently associated with inferior disease-free survival and ctDNA often preceding radiologic relapse (1). In broader localized NSCLC cohorts, Chaudhuri, Gale, and Chen each demonstrated that postoperative ctDNA can detect molecular relapse months before imaging, but these studies do not establish the cellular composition of MRD; rather, they define a clinical reservoir in which residual populations—including, but not necessarily limited to, persister-like states—may persist or evolve (2, 23, 24). Acquired resistance, in contrast, should be reserved for relapse states that display durable growth despite ongoing drug exposure and are often accompanied by stable genetic, epigenetic, or lineage-fixed changes. Shinozaki and colleagues make this distinction tangible: their EGFR-mutant organoid biobank captured resistant tumors with known genetic lesions, but also a subgroup with no canonical resistance alteration and a basal-shift phenotype, underscoring that clinically resistant disease can emerge through routes that are more stable than early persistence yet still not reducible to a single mutation (25). These categories should therefore remain analytically distinct: DTPs and cycling persisters are cell-state concepts, whereas MRD is a clinical detection state. Although these entities may overlap biologically, current assays do not allow direct one-to-one mapping between ctDNA-defined MRD and specific persister programs.

### 4.2 Temporal scale: a branching state map rather than a fixed linear sequence

Separating these terms clarifies their temporal and operational boundaries, but should not be interpreted as establishing a universal linear sequence. Acute cytoreduction, early persistence, clinically detectable MRD, and acquired resistance often occur in that approximate order at the population level, but individual clones may branch, overlap, regress, remain dormant, or progress through distinct routes. Early residual disease may contain reversible persisters, pre-existing resistant clones, genomically unstable populations, lineage-plastic states, or mixtures of these populations. These states may evolve in parallel, interconvert, or contribute unequally to later progression (13, 14, 26, 27). Oren’s lineage-tracing study is especially important here, because it shows that proliferative behavior under drug does not arise uniformly after treatment but reflects lineage-resolved fates detectable during the early persistence window (22). Nie’s work suggests that this phase may be experimentally interceptable in selected preclinical settings: interference with ACh/M3R signaling reduces DTP formation and delays relapse, implying that early persistence is not merely descriptive but biologically interceptable (21). The third phase is MRD maintenance, which is best defined clinically rather than morphologically. In the perioperative setting, NeoADAURA showed that neoadjuvant osimertinib, with or without chemotherapy, significantly improved major pathologic response relative to chemotherapy alone, supporting the clinical relevance of residual disease after EGFR-targeted therapy but not defining its cellular composition (28). In advanced disease, liquid-biopsy kinetics support the same idea from another angle: early decreases in ctDNA and circulating tumor cells on osimertinib are prognostic, suggesting that the biology of persistence begins well before radiographic progression becomes apparent (29). In some settings, this interval may include persister-derived populations, although its cellular composition is likely to vary by disease stage, treatment context, and assay modality. At overt progression, one or more surviving populations may expand and acquire stable genetic, epigenetic, bypass, or lineage-associated resistance features. However, the dominant progressive clone need not originate from a mitochondria-dependent DTP state. Importantly, the transition is neither instantaneous nor uniform. Gale and Chaudhuri showed that ctDNA can mark impending recurrence months before imaging in localized NSCLC, while Chen’s tumor-informed PROPHET assay extended this logic by demonstrating a median lead time of 299 days to radiologic recurrence (23, 24). These studies support the view that ctDNA-defined molecular recurrence can identify a clinically relevant interval between initial response and radiographic relapse, during which residual disease may persist; whether this interval uniformly represents active biological evolution, however, remains context dependent. This interval may provide the most relevant clinical window for testing persister-centered hypotheses.

### 4.3 Model systems: each model answers a different part of the persistence problem

The connection between clinical MRD and experimental persister biology is strongest when multiple layers of evidence converge: longitudinal ctDNA or imaging kinetics define the residual-disease interval; tissue, effusion, or resection specimens identify viable residual-cell phenotypes; single-cell and spatial assays localize those phenotypes within the tumor ecosystem; and patient-derived models test whether candidate programs are functionally required for survival under EGFR inhibition. Without such integration, MRD should be interpreted as a clinical state that may contain persister-like cells, partially adapted clones, genetically primed subclones, or mixed residual populations, rather than as a direct readout of one persister program (26, 30, 31). Instead, evidence is strongest when the model is matched to the biological question. Longitudinal clinical studies provide the clearest anchor for when residual disease exists in patients and why it matters prognostically (2). Patient-derived and matched translational models are most useful for testing whether candidate persistence programs remain plausible in a human disease context (32). Reductionist experimental systems, in turn, remain indispensable for defining mechanism, because they resolve lineage behavior, state transitions, and target dependence with a temporal precision that is not achievable in patients. These forms of evidence are complementary, but they differ in the kinds of inferences they can support. Dynamic cell-state systems are especially important for defining drug-tolerant persistence itself. *In vitro* lineage-tracing platforms, barcoding approaches, and single-cell analyses can distinguish cycling from non-cycling persisters, identify reversible versus stabilized adaptive programs, and test whether candidate dependencies truly sustain survival under continued EGFR inhibition (14). Their strength lies in mechanistic resolution rather than clinical generalizability. Put differently, these systems are best suited to explain how persistence is organized, but not to determine how frequently a given state occurs in human residual disease or whether it fully accounts for ctDNA-defined MRD. Patient-derived translational models occupy an intermediate position because they preserve more of the architecture of human disease while remaining experimentally tractable. Organoids derived from malignant pleural effusions, longitudinal organoid biobanks established after EGFR-TKI exposure, patient-derived xenografts, and paired on-treatment or post-progression specimens all help test whether proposed persistence programs remain relevant across treatment time and tissue context (33). Their value lies in strengthening biological plausibility in human-derived material. Even so, they do not by themselves establish prevalence, universality, or therapeutic effect size in patients. Clinical longitudinal studies provide the strongest anchor for MRD as a human disease state. Perioperative datasets, postoperative ctDNA studies, and serial plasma monitoring show that residual disease can remain clinically relevant below conventional radiographic detection and can precede overt relapse by months (34). These studies are therefore essential for defining the residual-disease window in real treatment trajectories. However, their strength is clinical rather than mechanistic: ctDNA-defined MRD does not by itself identify the cellular composition of the residual reservoir, nor does it specify whether that reservoir is composed mainly of classic DTPs, cycling persisters, partially adapted clones, or mixed populations that vary by stage and treatment context. This distinction matters for the way evidence is used throughout this Review. Taken together, these models support a practical interpretive rule for the rest of this Review: persister behavior is best defined in dynamic cell-state systems, MRD in longitudinal patient datasets, and acquired resistance in models that preserve evolutionary time and tissue context. Accordingly, evidence is interpreted by matching each model to the biological question it is best suited to address.

### 4.4 Interpretive framework for evidence use

**Figure 1:**
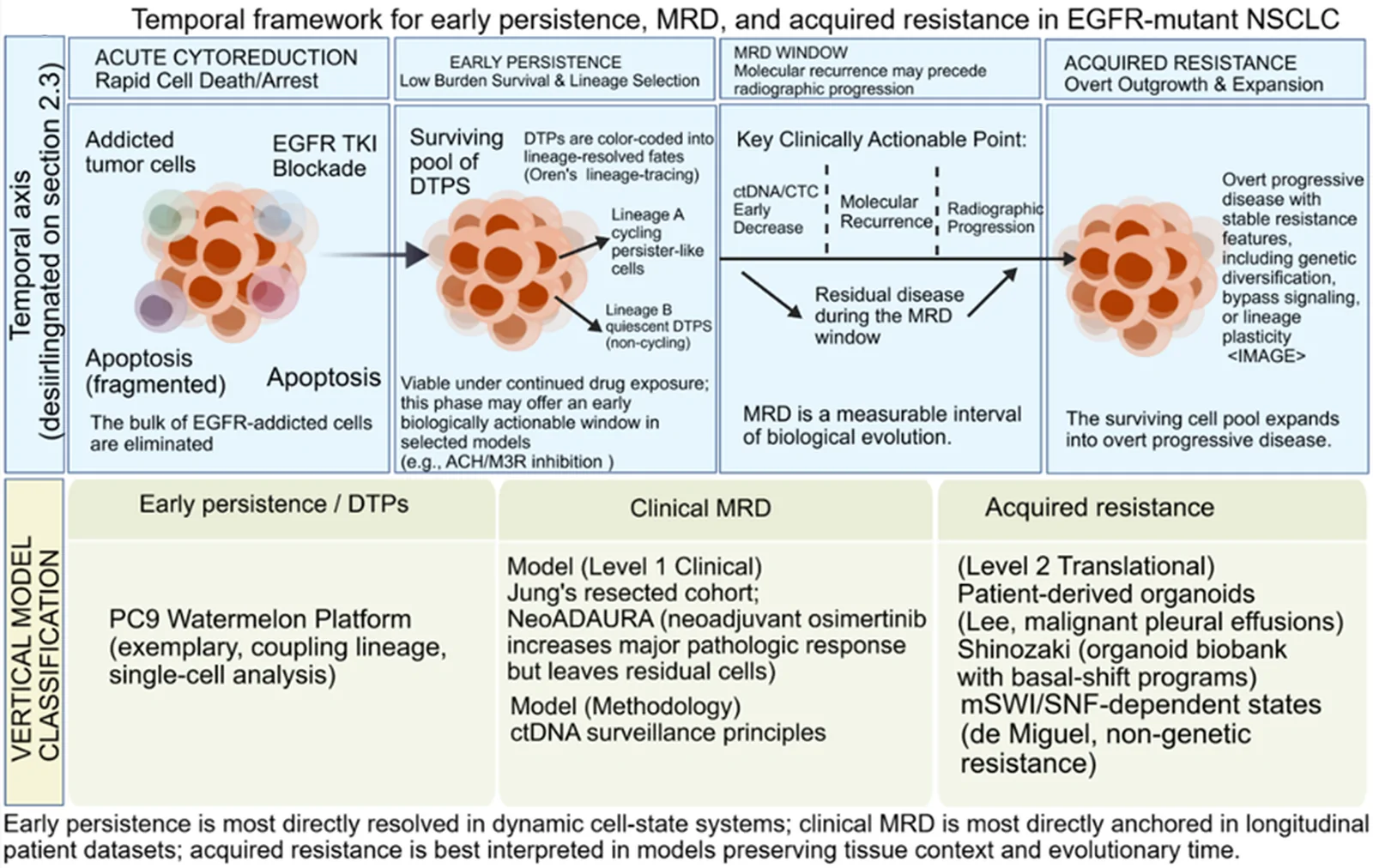
Proposed temporal and evidentiary framework for EGFR-TKI treatment failure. Early persistence, clinical MRD, and acquired resistance are shown as operationally distinct but potentially overlapping intervals. The scheme illustrates one possible trajectory and does not imply that all tumors pass through a common DTP-to-MRD sequence; parallel genetic, lineage, or epigenetic routes may dominate in individual tumors. The lower panel indicates the evidence systems most informative for each interval: dynamic cell-state systems for persister biology, longitudinal patient datasets for clinical MRD, and context-preserving translational models for acquired resistance. Created with biogdp.com.

Throughout the remainder of this Review, clinically anchored studies are used primarily to define disease state, timing, and prognostic relevance; patient-derived translational systems are used to test biological plausibility in human disease context; and mechanistic experimental studies are used to infer causal circuitry, without assuming direct equivalence among these forms of evidence. Evidence supporting individual components should not be interpreted as evidence that they form a single coordinated niche. To date, no study has simultaneously resolved tumor-cell mitochondrial dependence, CAF- or matrix-mediated support, immune function, and longitudinal residual disease within the same EGFR-mutant model or patient cohort. We therefore treat the mitochondrial niche as an organizing hypothesis, while restricting causal claims to the specific models in which each component was tested. The proposed temporal relationships among early persistence, clinical MRD, and acquired resistance, together with the evidence systems most informative for each interval, are illustrated in **Figure 1**.

## 5 Candidate tumor-intrinsic mitochondrial adaptations associated with EGFR-TKI persistence

### 5.1 Metabolic reconfiguration along a viability–stress continuum in selected preclinical persistence models

Available preclinical evidence suggests that a subset of sensitive EGFR-mutant cells that escape early killing can enter low-proliferation, survival-biased states in which glycolytic and cell-cycle programs are suppressed, while mitochondrial prosurvival programs are preserved or reweighted. In one of the earliest integrated studies of adaptive escape, Thiagarajan and colleagues showed that EGFR-TKI-treated escape cells were metabolically quiescent, with downregulation of glycolytic enzymes, induction of autocrine TGFβ2, and a bioenergetic configuration linked to mitochondrial priming rather than continued biomass production. Importantly, these cells were vulnerable to glutamine withdrawal and to disruption of mitochondrial antiapoptotic buffering, indicating that “quiescence” in this setting is an active bioenergetic choice rather than passive dormancy (35). A complementary line of evidence came from momcilovic and colleagues, who demonstrated that EGFR-mutant tumors remain highly dependent on glutamine utilization even when EGFR signaling is inhibited; combining erlotinib with glutaminase blockade induced a metabolic crisis *in vivo*, supporting the idea that carbon flow is rerouted rather than extinguished under targeted therapy (36). From an evidentiary standpoint, this view is supported mainly by mechanistic preclinical studies—including metabolomics, xenografts, and functional nutrient perturbation—rather than by direct profiling of patient MRD. Taken together, these findings support, in some EGFR-TKI persistence models, a shift from proliferation-associated metabolism toward survival-associated programs that preserve redox balance, mitochondrial integrity, and apoptotic restraint; however, this shift should be viewed as a continuum from adaptive downshifting to energetic collapse rather than as a strict binary distinction.

### 5.2 Mitochondrial quality control and redox buffering are candidate mechanisms sustaining this shift

If DTPs rely more heavily on mitochondrial function, mitochondrial quality-control pathways would be expected to become more important for their survival. This may help explain why mitochondrial quality-control pathways repeatedly emerge in persister models. Li and colleagues showed that drug-tolerant cells undergoing MAPK-targeted therapy switch from glycolysis to oxidative phosphorylation and depend on PINK1-mediated mitophagy to maintain mitochondrial homeostasis and redox balance; pharmacologic or genetic inhibition of this pathway markedly reduced tolerance and restored deeper treatment responses (37). Although not performed in EGFR-mutant NSCLC or under osimertinib pressure, this study provides a cross-context precedent that PINK1-mediated mitophagy can support DTP survival after targeted pathway inhibition; it should not be interpreted as disease-specific evidence that PINK1 is a recurrent dependency in EGFR-mutant NSCLC residual disease (37). More direct EGFR-mutant NSCLC evidence came from Hui Wang and colleagues, who described a cIGF1R/C-IGF1R axis that restrains Parkin-mediated VDAC1 ubiquitination, limits mitophagy, and pushes DTPs away from survival and toward apoptosis. In their system, activated mitophagy was not a generic stress marker; it was a functional gatekeeper of the DTP state, and restricting it reduced DTP emergence under EGFR-TKI treatment (38). Together, these studies are compatible with a model in which targeted therapy can increase mitochondrial stress and persister-like cells may rely more heavily on mitochondrial quality-control programs, and these studies suggest that mitophagy may help persister-like cells maintain oxidative metabolism while limiting mitochondrial damage and excessive ROS accumulation. This evidence is mechanistically strong, although direct validation in patient residual disease remains limited.

### 5.3 Iron and lipid peroxide handling may reveal liabilities of mitochondria-associated persister states

A maintenance metabolism centered on mitochondria inevitably changes how cells handle iron, lipid peroxidation, and oxidative injury. That is why ferroptosis-related vulnerabilities have become so informative in EGFR-TKI persistence models. Konishi and colleagues established osimertinib-tolerant PC9 persisters and found that, compared with parental cells, DTPs accumulated more intracellular ferrous iron and were markedly more sensitive to the GPX4 inhibitor RSL3; this death was rescued by iron chelation, indicating a bona fide ferroptotic liability (39). The importance of this observation lies not only in the therapeutic angle, but in what it says about the state itself: a persister cell can maintain viability under EGFR inhibition while simultaneously operating closer to an oxidative cliff. That interpretation is reinforced by Zhang and colleagues, who showed that EGFR-mutant lung adenocarcinoma cells with intrinsic or acquired EGFR-TKI resistance were more sensitive to ferroptosis inducers, and that vorinostat further enhanced ferroptotic killing through xCT downregulation (40). Wang and colleagues added a complementary perspective by showing that BCAT1-mediated branched-chain amino acid reprogramming is epigenetically induced under sublethal EGFR-TKI exposure and attenuates ROS accumulation, thereby facilitating tolerance and eventual resistance (41). Read together, these studies suggest that mitochondrial maintenance in persister models is closely linked to redox buffering, such that survival may depend in part on managing, rather than fully avoiding, oxidative stress. This redox-buffering dependence identifies a context-specific mechanistic vulnerability, but ferroptosis-based intervention should be considered cautiously because normal oxidative tissues, immune cells, and hematopoietic compartments may share related lipid-peroxide defenses. Any therapeutic translation would therefore require evidence of a persister-selective therapeutic window, biomarker-guided patient selection, and delivery or scheduling strategies that limit systemic ferroptotic toxicity (42–44). This subsection is supported primarily by convergent functional preclinical studies across independent models, with partial mechanistic convergence around redox handling and mitochondrial stress, but limited direct validation in human residual disease.

### 5.4 In available preclinical systems, mitochondrial adaptation appears to be more consistently linked to continued survival than to overt proliferative regrowth

**Figure 2:**
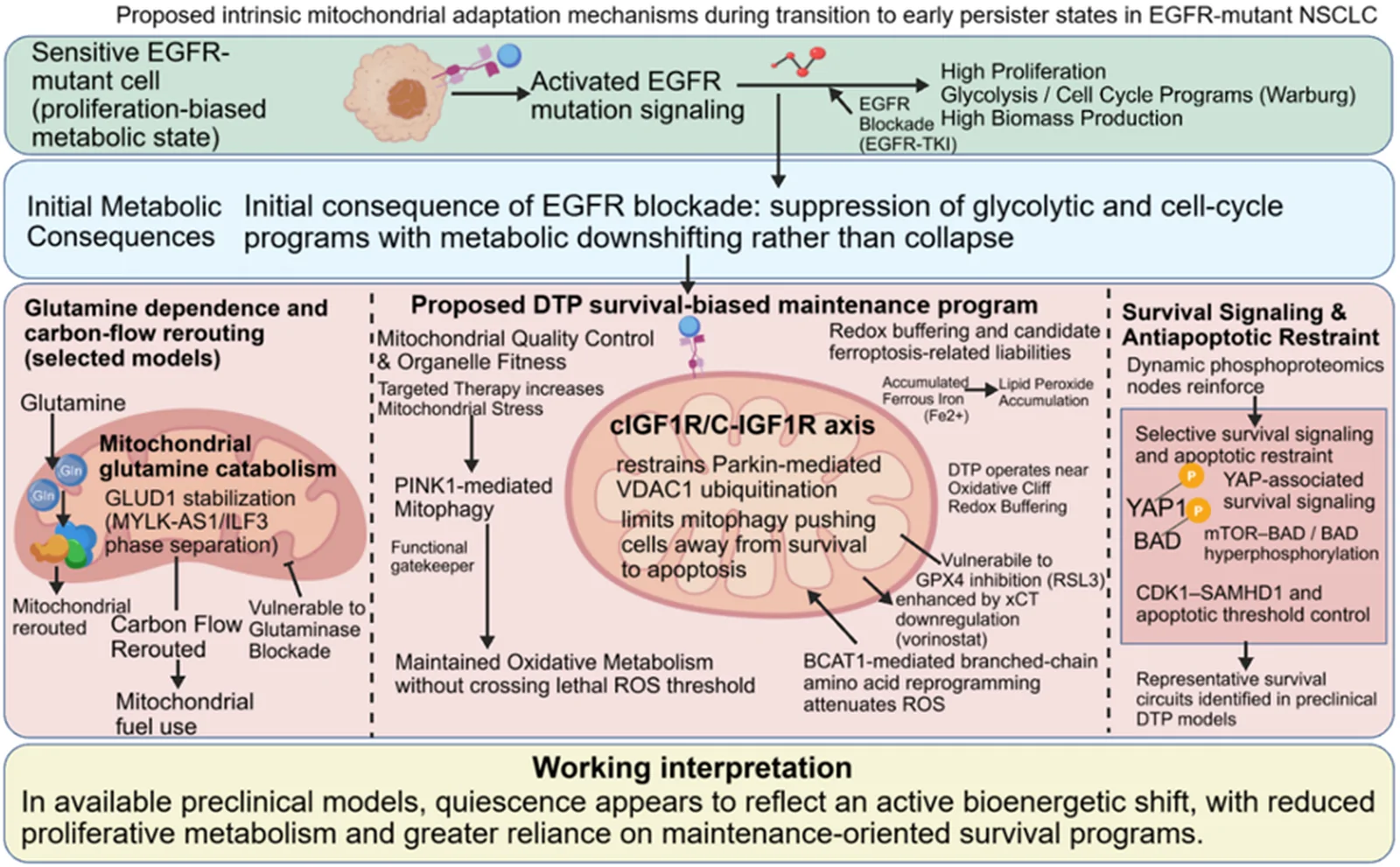
Proposed intrinsic mitochondrial adaptation programs associated with early persister survival in EGFR-mutant NSCLC. EGFR inhibition suppresses glycolytic and cell-cycle programs in sensitive EGFR-mutant cells, but in available preclinical systems this shift more often resembles metabolic downshifting than metabolic collapse. Early persister-like survival states are proposed to rely on maintenance-oriented programs that can include glutamine/carbon-flow rerouting, mitochondrial quality control, redox buffering, ferroptosis-related liabilities, and selective survival signaling with apoptotic restraint. Some modules are supported by direct mechanistic evidence in EGFR-mutant NSCLC, whereas others remain supportive cross-context persister mechanisms or integrative hypotheses. Created with biogdp.com.

Persister metabolism is better understood as a survival economy in which resources are redirected away from proliferation and toward redox control, mitochondrial fuel utilization, and apoptotic buffering. Qu and colleagues showed that the lncRNA MYLK-AS1 stabilizes GLUD1 through ILF3 phase separation, accelerates mitochondrial glutamine catabolism, and promotes EGFR inhibitor resistance *in vitro* and *in vivo*. This study is best interpreted as evidence that mitochondrial glutamine metabolism can become reinforced during established resistance, rather than as direct evidence of early DTP maintenance (45). Overall, the studies in this section identify recurrent but non-uniform mitochondrial and apoptotic dependencies in specific preclinical models; they do not establish a shared metabolic program across all DTP states or directly define the metabolism of patient MRD. A different but related view comes from dynamic phosphoproteomics. Hsu and colleagues found that osimertinib DTPs are characterized not by restoration of full proliferative signaling, but by selective reinforcement of survival circuits, including YAP1 activity, mTOR-BAD hyperphosphorylation, and CDK1-SAMHD1 signaling; inhibiting CDK1 impaired DTP growth and survival (46). Thus, in the systems discussed here, mitochondrial adaptation appears to support continued viability under drug pressure more consistently than overt proliferative regrowth. The strongest support here remains mechanistic and preclinical, because direct metabolomic profiling of patient MRD is still limited. Even so, across these preclinical studies, a recurring theme is that EGFR inhibition can suppress growth-associated metabolic programs while favoring survival-associated programs related to redox control, organelle fitness, and apoptotic restraint. The candidate tumor-intrinsic mitochondrial adaptation programs associated with early persister survival are summarized in **Figure 2**.

## 6 Proposed extrinsic components of a mitochondrial-niche model: how selected CAF states may buffer therapy-associated stress

### 6.1 From generic stromal rescue to defined paracrine bypass circuits

The earliest useful view of CAF-mediated EGFR-TKI failure was that fibroblasts provide soluble survival inputs when oncogenic EGFR is pharmacologically silenced. That model is now substantially sharper. In EGFR-mutant NSCLC, CAFs can drive gefitinib resistance by inducing EMT and upregulating ANXA2 through a dual HGF/IGF-1 program, showing that stromal rescue is not a vague trophic effect but a receptor-linked bypass architecture (47). Patient-derived analyses then added an important layer of selectivity: among 18 CAF isolates, only a subset robustly promoted EGFR-TKI resistance, and the common feature of these “resistance-promoting” CAFs was high HGF secretion, not uniformly elevated stromal cytokine output (48). More recently, the same paracrine principle has been extended to the osimertinib era. CAF-derived NRG1 was shown to enhance stemness, reduce apoptosis, and promote osimertinib resistance through HER3/AKT/NF-κB signaling, with *in vivo* confirmation in xenografts. Collectively, these studies establish ligand-specific CAF rescue in selected experimental systems, but do not demonstrate its causal coupling to tumor-cell mitochondrial dependence (49).

### 6.2 CAF heterogeneity matters because not all fibroblasts buffer therapy in the same way

A major advance in the field was the realization that CAFs are not uniformly resistance-promoting. In EGFR-mutant lung adenocarcinoma, CD200-positive CAFs unexpectedly increased the apoptotic effect of gefitinib and were associated with longer post-recurrence progression-free survival, indicating that some fibroblast states can sensitize rather than protect tumor cells (50). This heterogeneity was then functionally formalized in a living NSCLC CAF biobank: three CAF subtypes were identified, with subtype I and II fibroblasts showing the strongest protection against EGFR inhibition through HGF and FGF7, whereas subtype III fibroblasts were minimally protective and correlated with better clinical response (51). The newest layer of heterogeneity is even more operational. A single-cell-guided study identified CTHRC1-positive CAFs enriched in EGFR-TKI-resistant tumors; these fibroblasts drove tumor glycolysis through TGF-β/Smad3, while lactate fed back through H3K18 lactylation to further reinforce the CTHRC1-positive state, creating a self-sustaining resistance loop (52). Together, these studies indicate that fibroblast state, rather than CAF abundance alone, influences EGFR-TKI response. Because individual CAF populations may be protective, neutral, or sensitizing, these findings argue against unselected CAF depletion and favor state-specific biomarkers or functional-output interventions. At present, state-selective CAF targeting is more realistic as inhibition of defined outputs—such as HGF/MET, FGF7/FGFR, NRG1/HER3, TGF-β–matrix–YAP/FAK, kynurenine/AhR, or RhoA-dependent tunneling-nanotube formation—than as physical elimination of a CAF subtype; these approaches remain investigational.

### 6.3 Matrix and adhesion may convert stromal presence into a persistence-permissive mechanical context

CAF biology cannot be reduced to soluble factors, because fibroblasts also build the adhesive and mechanical context in which persister cells survive. The strongest current evidence comes from studies converging on YAP-centered mechanotransduction. Haderk and colleagues showed across patient-derived models, immunocompetent systems, and clinical specimens that a FAK-YAP axis promotes residual disease during targeted therapy, and that co-inhibition of this module suppresses drug-tolerant cells (4). Shi and colleagues then narrowed this principle to EGFR-mutant lung adenocarcinoma, identifying an ILK-SFK-YAP pathway that supports DTP viability, links EMT to persistence, and becomes more consequential in matrix-rich conditions; ILK-high tumors were also associated with receptor tyrosine kinase-independent resistance patterns in patient biopsies (53). This is best interpreted as mechanistically coherent evidence with emerging translational anchoring. CAFs, through matrix deposition and integrin-active microenvironments, likely contribute to the physical scaffold that permits these cytoprotective YAP programs to operate. These observations support viewing the niche as not only biochemical but also, at least in part, biomechanical. This matters for a mitochondrial-niche framework because mechanotransduction and mitochondrial fitness are tightly coupled: a cell that has shifted away from proliferative metabolism still requires adhesion-dependent survival signaling to remain viable under chronic drug stress.

### 6.4 The niche may acquire a more explicit metabolic dimension when selected CAF states buffer stress products or accept damaged organelles

**Figure 3:**
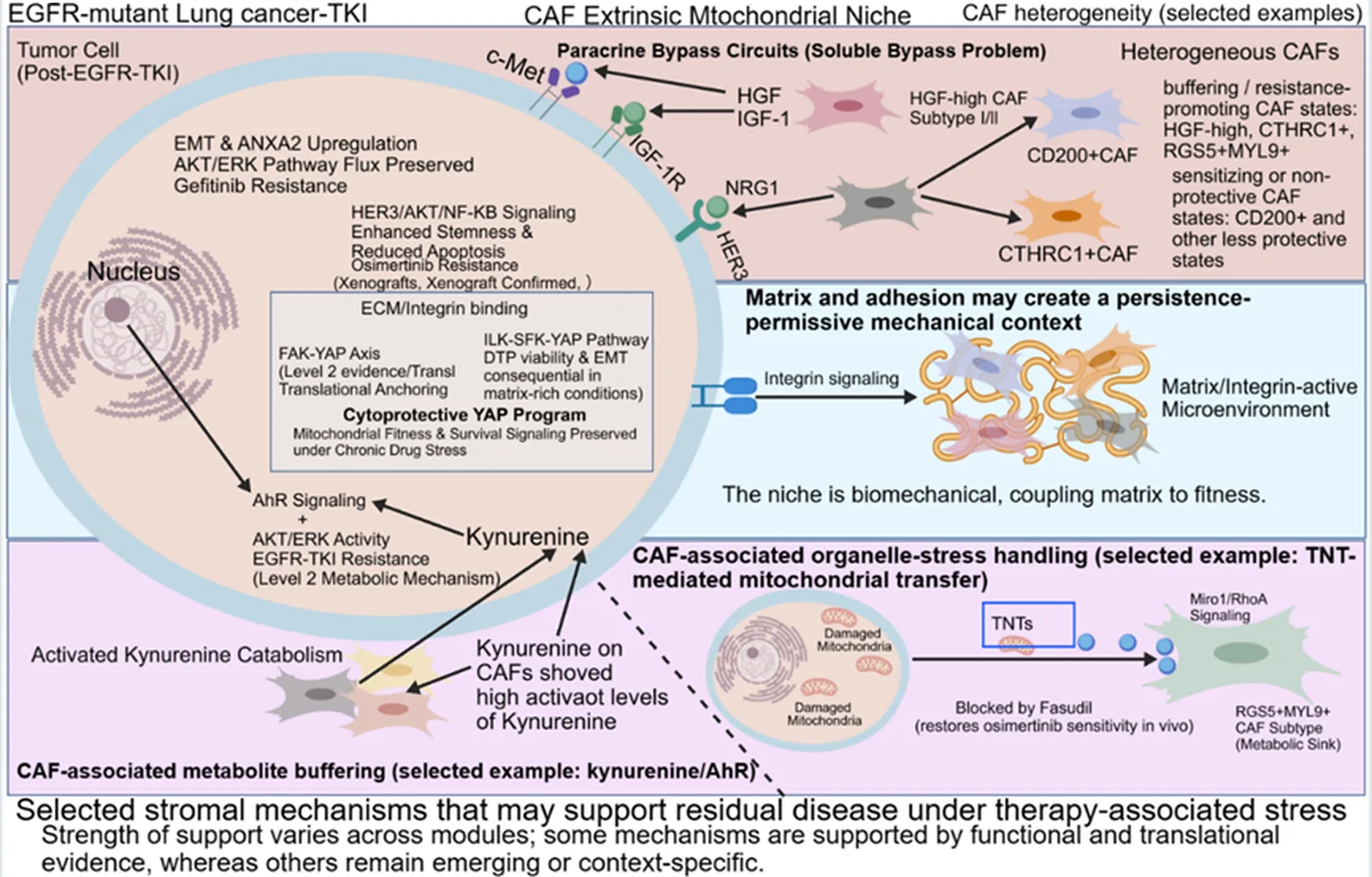
Candidate CAF-mediated components of the mitochondrial-niche model. Selected CAF states may support EGFR-TKI persistence through ligand-dependent bypass signaling, matrix-associated mechanotransduction, or metabolite and organelle-stress handling. These mechanisms were identified in separate systems and are not assumed to coexist in the same tumor. CAF states may be protective, neutral, or sensitizing, and evidence strength differs across modules. Created with biogdp.com.

Experimental studies suggest that CAF-associated support may include paracrine signaling, matrix-associated mechanics, metabolite exchange, and organelle-stress handling, but these functions should be viewed as non-mutually exclusive modules rather than as a fixed hierarchy. CAFs from lung-cancer models were shown to produce kynurenine through activated tryptophan catabolism; in experimental systems, this metabolite activated AhR signaling in tumor cells, sustained AKT/ERK activity, and promoted erlotinib/gefitinib resistance, while AhR inhibition improved drug response (54). Because these CAFs were not specifically derived from osimertinib-treated EGFR-mutant residual disease, this mechanism should be treated as supportive preclinical evidence for CAF metabolic buffering rather than as disease-state-specific evidence. More recently, a study identified an RGS5^+^MYL9^+^ CAF population with potential clinical relevance was recruited to EGFR-TKI DTP niches, formed tunneling nanotubes through Miro1/RhoA signaling, and accepted damaged mitochondria from tumor cells. In one recent study, these fibroblasts acted as ‘metabolic sinks,’ and pharmacologic inhibition of mitochondrial transfer with fasudil restored osimertinib sensitivity *in vivo*; however, the prevalence of this mechanism across clinical residual-disease settings remains unknown (7). These findings identify metabolite exchange and mitochondrial disposal as candidate CAF-mediated stress-buffering mechanisms, but their prevalence in patient MRD and coordination with immune remodeling remain unknown. The kynurenine/AhR mechanism should be regarded as supportive preclinical evidence for metabolic buffering, whereas RGS5^+^MYL9^+^ CAF-mediated mitochondrial transfer provides a mechanistically integrated but still single-study proof-of-concept. Accordingly, CAF-directed intervention is best framed at present as biomarker-guided targeting of functional outputs rather than as clinically established state-selective CAF depletion. These findings support the possibility that in some settings, residual survival after EGFR inhibition is shaped not only by tumor-cell adaptation but also by stromal handling of therapy-associated stress. The candidate CAF-mediated components of the mitochondrial-niche model are summarized in **Figure 3**.

## 7 Residual disease may sustain stress-associated inflammation without consistent productive antitumor immunity

### 7.1 The ‘immune-cold’ state of EGFR-mutant NSCLC may be actively reinforced rather than simply passively inherited

A useful starting point is that EGFR-mutant NSCLC often exhibits an immune-excluded phenotype, and available evidence suggests that oncogenic EGFR signaling can contribute to maintaining this state. In EGFR-mutant models and paired clinical samples, EGFR pathway activity suppresses tumor-cell MHC-I expression through MEK–ERK signaling, whereas EGFR-TKI treatment restores MHC-I, increases CD8^+ infiltration, and is accompanied by PD-L1 induction (55). In parallel, EGFR signaling drives a second layer of exclusion by increasing CCL22 and recruiting effector Tregs while reducing IRF1-dependent CXCL10 and CCL5, thereby limiting CD8^+ T-cell trafficking. In syngeneic EGFR-mutant models, EGFR blockade decreased intratumoral Tregs and improved the activity of PD-1 blockade (56). Taken together, these studies support an overall immune-low tendency in many EGFR-mutant tumors, but this should not be treated as a uniform baseline state. Some EGFR-mutant tumors are inflamed before treatment, and baseline immune context varies by mutation subtype, PD-L1 status, co-mutations, smoking history, and tissue site. These baseline differences may constrain—but do not uniformly determine—subsequent immune clearance after EGFR-TKI treatment.

### 7.2 EGFR-TKI treatment can ignite inflammation, but the response is heterogeneous, transient, and rarely self-sustaining

Once EGFR is inhibited, the tumor does not remain immunologically static. In murine EGFR-driven lung tumors, tumor regression after either erlotinib treatment or oncogene withdrawal was sufficient to recruit inflammatory T cells and promote macrophage maturation (57). A second study extended this to the transcriptional level in cell lines and in eight paired patient biopsies obtained before and after short-term EGFR-TKI exposure (58). The response was heterogeneous: IFNγ enrichment correlated with time to progression, but the cohort was too small to define robust host or tumor predictors. T-cell signatures increased in three of four patients with longer time to progression, whereas the shorter-response group showed mixed changes. Yet the response is highly variable. In 138 paired biopsies obtained before and after EGFR-TKI treatment, PD-L1-high tumors became more frequent after therapy, but both CD8^+ and FOXP3^+ TIL densities generally fell, suggesting that inflammatory remodeling is not equivalent to durable immune engagement (59). More recently, transcriptomic profiling of paired baseline and resistant tumors found that about one-third of post-TKI samples acquired a “hotter” phenotype, particularly among L858R tumors, again underscoring heterogeneity rather than a uniform shift toward immune competence (60). These findings provide clinically and translationally relevant support for the idea that EGFR-TKI treatment can generate stress inflammation, but that the resulting inflamed state is often partial, unstable, and biologically decoupled from complete tumor elimination.

### 7.3 In some settings, treatment-associated inflammation may be redirected into escape circuitry rather than effective killing

Why, then, does treatment-associated inflammation so often fail? One answer is that EGFR inhibition can activate innate immune escape programs at the same time that it induces danger signaling. In EGFR-mutant NSCLC cells, EGFR-TKI exposure increases tumor-cell CD24 and accelerates cfDNA release from dying cells, which activates type I IFN signaling in monocytes through STING-dependent pathways. Rather than simply enhancing immunity, this state favors an innate checkpoint configuration consistent with immune escape (61). A separate coculture-based study reached a similar conclusion through a different route: early during EGFR-TKI treatment, CXCL10 levels rose in the tumor microenvironment and, through CXCR3 on tumor cells, activated Src, NF-κB, and HIF-1α, thereby supporting persistence via both autocrine and paracrine signaling (62). In selected models, treatment-associated inflammatory signals are redirected toward tumor-cell survival or innate-checkpoint reinforcement rather than effective immune clearance. These mechanisms remain insufficiently linked to prospective MRD kinetics in patients. Although CXCL10/CXCR3-related circuits are pharmacologically interruptible in principle, broad blockade could also impair desired T-cell recruitment; tumor-cell-restricted downstream nodes may therefore be more practical than unselected chemokine inhibition. Even so, these studies explain why “inflamed” and “immune effective” should not be treated as synonyms in EGFR-TKI-treated disease.

### 7.4 Myeloid-dominant states may limit the conversion of treatment-associated inflammation into cytotoxic immunity

One of the clearest mechanistic explanations for incomplete immune clearance in residual-disease models comes from the myeloid compartment. In an immunocompetent EGFR-mutant mouse model, osimertinib-induced tumor regression required CD8^+ T cells, yet advanced tumors enriched for M2-like TAMs failed to mount the same T-cell activation and became refractory to regression. In that mouse model, systemic STING agonism with MSA-2 reprogrammed macrophages and improved osimertinib-associated antitumor immunity (63). However, this should be viewed as preclinical proof-of-concept, because STING agonists have not yet established durable clinical efficacy in solid tumors and systemic activation can produce toxicity or context-dependent immune suppression. This is a particularly important result because it shows that the limiting factor is not simply tumor-cell signaling but the immune ecology of the residual niche. Clinical observations point in the same direction. In EGFR-mutant NSCLC treated with nivolumab after EGFR-TKI failure, T790M-negative tumors showed higher PD-L1 expression, greater CD8^+ TIL density, and better outcomes than T790M-positive tumors, implying that immune responsiveness after targeted therapy depends on the biological route by which resistance evolves (64). Taken together, these data suggest that macrophage-dominant suppression is an important contributor to failed immune clearance after EGFR-TKI treatment, with the magnitude of this effect likely varying by resistance trajectory and microenvironmental context. In other words, stress inflammation is not enough when the residual niche remains myeloid-restricted and T-cell permissive only in a minority of cases.

### 7.5 Residual disease leaves behind selectable immune surface programs and inflammatory memory

One reason immune failure may be structured rather than purely stochastic is that persister-like and resistant cells can acquire surface programs and transcriptional memory states that are immunologically consequential. CD70 is one such program. In EGFR-TKI-refractory tumors, particularly those with EMT-associated resistance, CD70 is markedly increased in paired clinical samples and is associated with worse survival; functional targeting with CD70-directed CAR-T or CAR-NK cells suppresses resistant and persister-like populations in preclinical models (65). A second example is MUC1-C. Recent work shows that osimertinib resistance can be stabilized by a STAT1-centered inflammatory memory program orchestrated by MUC1-C at enhancer-like regulatory elements, thereby promoting resistance across MET-amplified and EGFR compound-mutant contexts. Importantly, this memory state is therapeutically tractable in preclinical systems with a MUC1-C-directed ADC (66). A complementary 2025 study adds that EGFR TKIs themselves can expose the hypoglycosylated TnMUC1 antigen by suppressing C1GALT1, thereby improving recognition by TnMUC1 CAR-T cells in xenografts and PDXs (67). These examples show that persister and resistant states can acquire immune-surface or inflammatory-memory programs, but their coupling to mitochondrial or CAF biology remains unproven. Such programs may be preclinically targetable, but targetability is not equivalent to clinical tractability. For CD70, MUC1-C, TnMUC1, and related surface targets, development will require antigen-density thresholds, normal-tissue mapping, immune-cell toxicity assessment, and validation in immune-competent or clinically relevant systems.

## 8 Candidate biomarkers and translational requirements for testing the mitochondrial-niche framework

### 8.1 Tumor-cell biomarkers should aim to identify tumors with a higher propensity for adaptive persistence before overt resistance emerges

A clinically useful biomarker must have a defined intended use, analytically reproducible measurement, a prespecified cutoff, and prospective evidence that biomarker-guided intervention improves a clinically relevant outcome. Most candidates discussed below currently demonstrate biological plausibility or preclinical functional relevance rather than clinical qualification. Two types of evidence are especially informative. First, in an *in vivo* EGFR-mutant patient-derived xenograft, single-cell profiling revealed that DTP tumors preserved baseline genomic architecture yet contained a rare pretreatment DTP-like subpopulation with intermediate activation of pathways later amplified in drug tolerance, suggesting that persister competence may be detectable before therapy begins (68). Second, EGFR-TKI exposure itself can generate measurable surface and metabolic markers. DPP4 was upregulated in EGFR-TKI-induced persister models, and combined osimertinib–sitagliptin reduced residual tumor burden *in vivo*, supporting DPP4 as a preclinical candidate rather than a clinically validated selection biomarker (6). A multi-model DTP screen identified recurrent sensitivities to TEAD, BRD4, AURKB, and MEK inhibition, providing candidates for further validation but not an assay-ready biomarker panel (69). In practical terms, Tumor-cell biomarkers may be prioritized into three practical categories: clinically anchored markers linked to patient outcome, functionally validated markers supported by *in vivo* or patient-derived models, and exploratory discovery signatures that remain hypothesis-generating. The real goal is not to generate longer marker lists, but to define a compact panel that captures pretreatment persister propensity, on-treatment adaptation, and actionable vulnerability in the same patient trajectory.

### 8.2 Stromal biomarkers are most useful when they capture fibroblast state, not fibroblast abundance

For the extrinsic niche, simply quantifying “CAF-rich” tumors is unlikely to be informative enough. The more discriminating approach is to identify fibroblast states that correlate with drug buffering. A strong early example came from podoplanin-positive CAFs. In EGFR-mutant lung adenocarcinoma, coculture with podoplanin-expressing CAFs maintained ERK signaling under gefitinib, reduced EGFR-TKI sensitivity, and, importantly, high podoplanin expression in CAFs from surgical specimens was associated with a lower response rate to EGFR-TKIs in recurrent disease (70). This already suggested that stromal biomarkers can be both mechanistic and predictive. A more recent clinical refinement makes this far more actionable. In a 2024 study of EGFR-mutated lung adenocarcinoma treated with osimertinib, solid-predominant histology and high podoplanin expression in CAFs were each independently associated with shorter progression-free survival, and tumors harboring both features exhibited higher HGF expression in independent datasets (71). These findings support biological and prognostic relevance but do not establish podoplanin as a predictive treatment-selection biomarker. Reproducible clinical use will require standardized CAF definitions, spatial quantification, sampling across treatment stages and metastatic sites, and prespecified antibody clone, staining platform, compartment annotation, scoring threshold, and inter-observer reproducibility. For future studies, stromal biomarker programs should move beyond bulk fibroblast markers and instead ask which CAF states reproducibly track with HGF-rich, matrix-active, or metabolic-buffering niches during the MRD window.

### 8.3 Immune biomarkers should reflect residual immune function, not just checkpoint abundance

The immune dimension of the niche is often oversimplified as PD-L1 status. In EGFR-mutant NSCLC, that is insufficient—but not irrelevant. Strong tumor PD-L1 expression was associated with poorer EGFR-TKI efficacy and was enriched in *de novo* resistance, often in tumors with concurrent CD8 positivity, suggesting that an “inflamed” phenotype can still be clinically ineffective (72). Plasma-based immune markers may add complementary information. In a cohort of EGFR-mutant NSCLC treated with first-line EGFR-TKIs, high baseline soluble PD-L1 independently predicted shorter progression-free survival, and high pretreatment or on-treatment sPD-L1 was associated with lower response rates (73). These findings argue that immune biomarkers in this setting should include both tissue and circulating compartments. T-cell receptor readouts may be even more informative than static checkpoint measurements. In the ADJUVANT-CTONG1104 trial, specific TCRβ rearrangements were associated with EGFR genotype and with favorable overall survival, particularly in the adjuvant gefitinib setting (74). These findings support evaluation of both tissue and circulating compartments, but differences in assay platform, sampling time, and cutoff currently limit cross-study comparison. T-cell receptor readouts may capture immune state more deeply than static checkpoint measurements, but they remain technically and analytically demanding. A related retrospective analysis generated a TCR-sequence model associated with prognosis and possible differential benefit from adjuvant EGFR-TKI therapy, requiring independent prospective validation (75). No immune-state assay described here is currently standardized for selecting EGFR-mutant patients for residual-disease intervention. The larger point is that residual immune inefficiency should be measured as a functional immune landscape, not as checkpoint intensity alone.

### 8.4 Dynamic monitoring should focus on residual signal, not only radiographic response

Because the proposed framework concerns a temporally evolving residual-disease state, candidate biomarkers should be assessed longitudinally. Liquid biopsy can track residual tumor-derived signals over time, but it does not directly resolve niche composition, spatial organization, or mitochondrial state. In patients receiving osimertinib after prior EGFR-TKIs, combined monitoring of CTCs and ctDNA showed that persistent liquid-biopsy positivity tracked with inferior outcomes, supporting the use of blood-based monitoring as an early surrogate of inadequate biologic control (29). Prospectively integrated ctDNA kinetics from SWOG S1403 extended this principle: complete clearance of mutant EGFR ctDNA at 8 weeks was associated with markedly longer progression-free and overall survival than persistent ctDNA, and ctDNA clearance outperformed RECIST response as a predictor of long-term benefit (31). Dynamic biomarker design should include driver-ctDNA clearance, residual ctDNA persistence, and CTC-state shifts rather than relying on RECIST response alone; however, ctDNA clearance may reflect tumor-volume reduction or altered DNA shedding rather than collapse of a persister ecology. Prospective studies should therefore interpret ctDNA kinetics with baseline tumor burden, disease site, assay sensitivity, and tissue or cellular validation when available (29, 31). These assays are among the most clinically informative biomarker approaches when embedded prospectively in trials. For future EGFR-mutant MRD studies, the ideal design would align early liquid-biopsy kinetics with on-treatment tissue profiling, allowing investigators to ask not only whether a patient is responding, but whether the residual ecosystem is collapsing—or merely being compressed below the threshold of imaging. Notably, ctDNA persistence should be interpreted as a residual-disease signal rather than a direct surrogate for any specific persister program.

### 8.5 Methodological rigor will determine whether “niche biology” becomes a field or remains a metaphor

**Figure 4:**
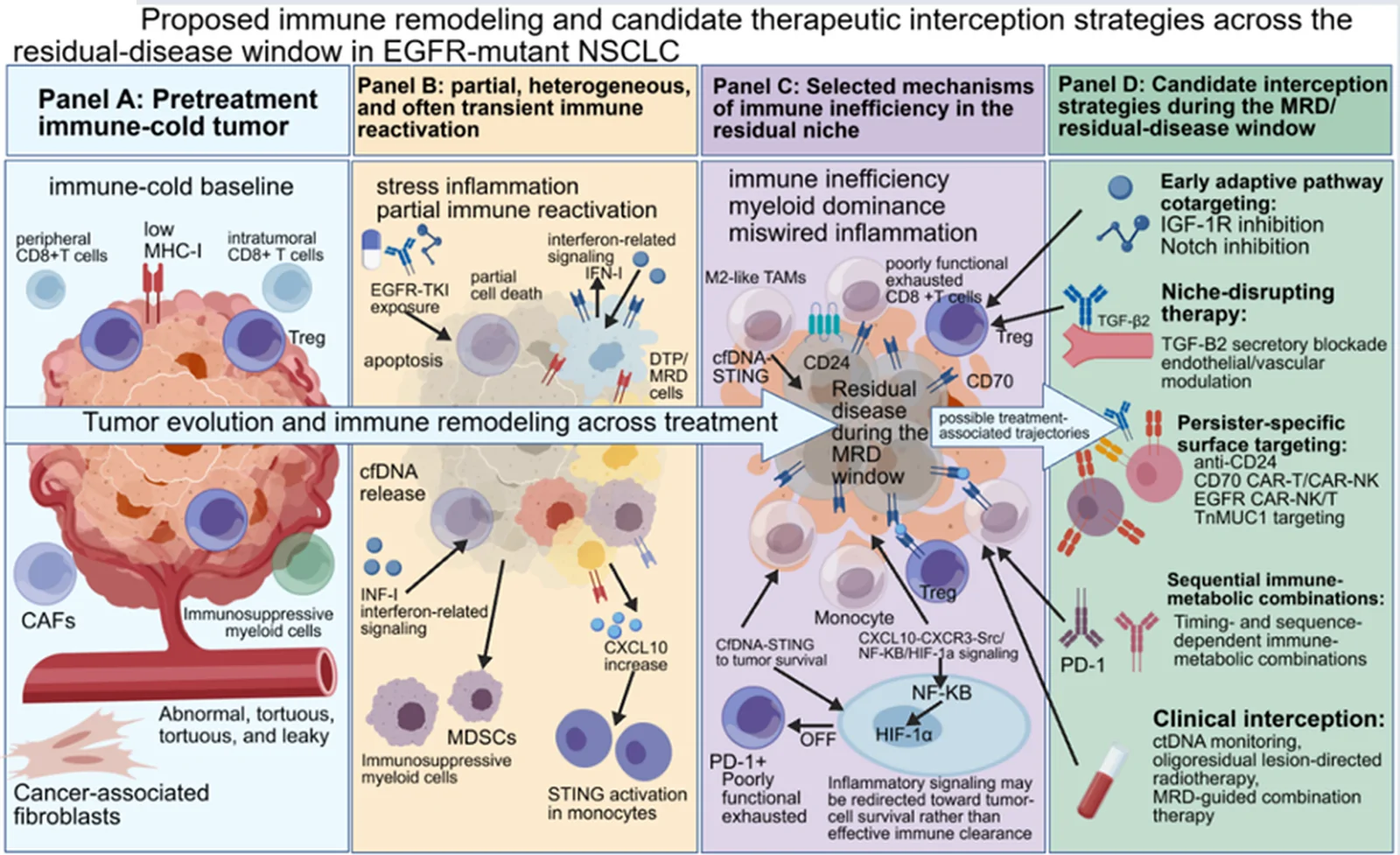
Proposed immune remodeling and candidate interception strategies across the residual-disease window in EGFR-mutant NSCLC; before treatment, EGFR-mutant NSCLC often displays an immune-cold baseline characterized by limited effective cytotoxic infiltration and suppressive stromal-myeloid features. Early during EGFR-TKI treatment, stress-associated inflammation and interferon-related signaling may emerge, but this reactivation is frequently partial, heterogeneous, and transient. In selected residual-disease settings, immune inefficiency may persist through myeloid dominance, miswired inflammatory signaling, and residual-cell surface or memory programs, rather than through complete immune silence. Candidate interception strategies therefore include pathway cotargeting, niche-disrupting interventions, persister-surface targeting, and clinical monitoring or trial-enabling approaches; however, these strategies differ substantially in evidence level and clinical maturity. Created with biogdp.com.

The niche concept will only matter if it is matched by methods capable of resolving cell state, spatial context, and temporal evolution together. Single-cell clinical sampling already shows what becomes visible once this standard is applied. In a landmark study of 49 metastatic lung cancer biopsies obtained before and during targeted therapy, residual disease was characterized by an alveolar-regenerative program, whereas progressive disease displayed immunosuppressive cell states and distinct tumor-cell pathway activation; notably, these features also predicted outcomes in independent cohorts (26). More recently, integrated single-cell RNA-seq plus spatial transcriptomics in EGFR-mutated lung cancer showed that BCL2L1/BCL-XL is enriched in treatment-shaped tumor states and spatially heterogeneous regions, with functional inhibition delaying or overcoming EGFR-TKI resistance (76). An additional methodological bridge comes from composite resistance scoring. Using resistant stem-cell-associated genes, Xie and colleagues built an R-index that was higher in resistant cell lines, mouse models, and relapsed patient cohorts, and associated elevated scores with shorter relapse time and an MDSC-rich microenvironment (77). This kind of integrative model is valuable because it converts complex biology into a testable patient-level hypothesis. The strongest studies will combine pretreatment and on-treatment tissue, single-cell or spatial profiling, serial liquid biopsy, and functional validation, but this should be treated as an aspirational benchmark rather than a minimum requirement for every trial. A pragmatic design would apply serial ctDNA to all patients, collect baseline tissue, obtain early on-treatment biopsies when feasible, and reserve single-cell, spatial, or patient-derived functional assays for enriched subsets. This tiered approach preserves biological resolution while acknowledging tissue access, patient burden, cost, turnaround time, sampling bias, and batch effects. The proposed evolution of immune remodeling and the principal candidate interception strategies across the residual-disease window are summarized in **Figure 4**.

## 9 Candidate therapeutic-interception strategies for the early persistence and residual-disease window

### 9.1 Intercept tumor-intrinsic tolerant circuitry before it hardens into stable resistance

A central implication of the persister model is that the most effective combination may not be the one that best suppresses overtly resistant clones, but the one that prevents a viable tolerant reservoir from ever stabilizing. Two studies make this point with unusual clarity. In AXL-low EGFR-mutant models, osimertinib induced a FOXA1–IGF-1R survival program; importantly, transient IGF-1R inhibition combined with continuous osimertinib eradicated tumors in cell-line and patient-derived xenografts and prevented regrowth after drug withdrawal (78). In selected biomarker-defined settings, early cotargeting of adaptive survival pathways may be more effective than intervention after overt progression, but this premise requires prospective testing. The available studies provide preclinical proof-of-concept for a time-limited intervention strategy, but they do not establish clinical efficacy, an acceptable therapeutic index, or the optimal intervention window. Translation will require prespecified early-treatment biomarkers and pharmacodynamic confirmation of target engagement (20, 79). A second example comes from Notch biology. Osimertinib DTP cells upregulated NOTCH1 and downstream target genes, and addition of a γ-secretase inhibitor impaired DTP persistence *in vitro* and *in vivo* (80). These data support the hypothesis that, in selected settings, early and time-limited cotargeting of adaptive survival pathways may be more rational than chronic empiric intensification. The logic is not to treat every latent bypass axis indefinitely, but to strike those circuits that are selectively needed during the transition from acute drug stress to residual viability. In evidence terms, this is strong Level 2 preclinical evidence: the studies use functional perturbation and *in vivo* validation, but prospective biomarker-guided clinical trials are still lacking. These studies provide proof-of-concept that adaptive survival circuitry can be targeted during the early residual-disease phase. Whether such strategies will define a clinically useful intervention window remains to be tested prospectively.

### 9.2 Niche-disrupting combinations may need to target the ecology of residual disease, not only the tumor cell

If a defined extrinsic component is shown to be necessary for a particular residual state, combination therapy could target that component alongside EGFR inhibition. One emerging route is to block therapy-induced stromal signaling. In 2025, osimertinib was shown to activate a TGF-β2-dependent secretory program that promotes lung adenocarcinoma progression, suggesting that some of the pro-survival niche is actively *generated by treatment itself* (81). A second route is to target the vascular-immune compartment. In a preclinical EGFR-mutant setting, osimertinib improved the tumor immune microenvironment and enhanced the antitumor effect of bevacizumab by reducing PD-L1 expression in tumor-associated endothelial cells (82). These studies provide separate preclinical rationales for targeting stromal secretion and vascular-immune signaling, but they do not validate a unified niche-disruption strategy. Because CAF states may be protective, neutral, or sensitizing, broad stromal depletion could be ineffective or harmful. Clinical development should require evidence that the targeted program is present, spatially accessible, functionally associated with residual survival, and measurable by a prespecified pharmacodynamic assay. TGF-β-directed strategies should also be framed cautiously because of limited clinical efficacy and historical cardiac or systemic toxicity concerns. By contrast, a niche-based strategy starts from a more specific question: what nonmalignant compartment is preserving residual viability after initial response, and when is that support most vulnerable? At present, these combinations should be viewed as niche-disrupting hypotheses with mechanistic rationale, rather than as clinically validated residual-disease strategies.

### 9.3 Persister cells may represent a distinct target population with their own surface vulnerabilities

Persister-associated surface programs may create therapeutic targets distinct from pretreatment tumor dependencies. In EGFR-mutated lung cancer, CD24 increased across drug-responsive cells, DTPs, and resistant cells after third-generation EGFR-TKI exposure; genetic depletion or the anti-CD24 antibody ATG-031 enhanced macrophage-mediated phagocytosis and improved tumor eradication in xenografts, spontaneous models, and PDX systems (83). This is notable because it converts a residual immune-evasion program into a therapeutic entry point. A related but distinct strategy is cell therapy. EGFR-directed CAR NK and CAR T cells showed activity against EGFR-mutant drug-tolerant persister cells (DTPCs) and drug-resistant cells, with CAR NK cells showing particular promise; efficacy was further enhanced by continued EGFR-TKI exposure or TGF-β pathway blockade (84). The narrower conclusion is that persister-enriched surface states may provide therapeutic entry points beyond EGFR kinase inhibition. Current support is preclinical and translational rather than clinical, and development must address antigen heterogeneity and loss, on-target/off-tumor toxicity, immune-cell fratricide, trafficking into residual lesions, and the duration of target expression (65, 85). The strongest support here is Level 2 translational evidence. The translational promise is clear, but patient-selection rules, safety considerations, and the durability of persister-specific targeting remain unresolved.

### 9.4 Immune-metabolic combinations will depend on timing and sequence, not simply on adding checkpoint blockade

Current data do not exclude immune-based interception of EGFR-mutant residual disease, but they suggest that timing and sequence may be critical determinants of benefit. In syngeneic EGFR-mutant lung tumors, EGFR-TKI treatment induced CD8^+ T-cell responses that were further amplified by sequential dual PD-1/VEGFR2 blockade, whereas simultaneous triple blockade did not produce the same benefit (86). Clinical experience reinforces the need for restraint. In a phase IB study, afatinib plus pembrolizumab showed only modest activity after progression on prior EGFR TKI, although on-treatment immune profiling did detect increases in NK/NKT cells and decreases in exhausted CD8^+ cells (87). In other words, indiscriminate TKI–ICI pairing can produce biologic movement without reliably translating into durable disease control. The implication for a mitochondrial-niche framework is that immune-metabolic combinations should be designed around state transition, not around late-line desperation. Rational sequencing, vascular co-targeting, and biomarker-enriched cohorts are more likely to succeed than broad, unselected checkpoint addition. These findings support a sequencing hypothesis: checkpoint or vascular intervention may be more productive after initial EGFR-TKI debulking than at treatment start. Rational sequencing, vascular cotargeting, and biomarker-enriched enrollment should be prospectively compared with broad, unselected checkpoint addition. Such trials must account for schedule-dependent pulmonary, hepatic, vascular, and immune toxicities and should use fixed early landmarks and prespecified biomarker criteria rather than *post-hoc* patient selection.

### 9.5 Clinical trial architecture may need to move intervention earlier and treat residual sites as biologically important

To test whether early interception improves outcome, trials must prospectively define the residual-disease population, intervention time point, biomarker, and comparator. FLAURA2 and LAURA did not measure persister states, MRD composition, or mitochondrial-niche biology; they are cited only as clinical examples of treatment intensification earlier in the disease course, and their efficacy should not be interpreted as evidence that benefit resulted from elimination of a persister ecology (88, 89). Residual-site control may also matter more than is often appreciated. A recurrence-pattern analysis of metastatic EGFR-mutant NSCLC treated with osimertinib found that a meaningful subset of patients had oligoresidual disease at maximal response, providing a rationale for local consolidative therapy (89). That concept has now been prospectively tested: in a phase II study, osimertinib followed by consolidative radiotherapy to persisting lesions after 8 weeks was well tolerated and showed promising efficacy (90). Future interception trials should incorporate prespecified biomarker eligibility, standardized baseline and on-treatment sampling, explicit rules for local versus systemic treatment of persisting sites, an appropriate randomized comparator, and endpoints encompassing assay-defined MRD clearance, radiographic progression, survival, toxicity, and patient-reported outcomes. PFS improvement alone cannot establish collapse of a persister ecology.

## 10 Controversies and future directions: what is already solid, and what should still be treated as hypothesis

### 10.1 One strong conclusion is that residual disease is real, biologically structured, and heterogeneous

Preclinical residual-disease models indicate that post-EGFR-TKI survivors can be transcriptionally and functionally heterogeneous rather than featureless. A 2024 PDX-based study showed that osimertinib-tolerant residual cells were transcriptionally heterogeneous, that DTRC-like states could already be detected before treatment, and that ASCL1-driven plasticity promoted tolerance only in permissive cellular contexts rather than uniformly across tumors (91). A 2025 study then added an orthogonal layer of heterogeneity by identifying GSTA1 as a tolerance-conferring determinant; the available report indicates that elevated GSTA1 promoted osimertinib degradation and linked redox-detoxification biology to the DTP state (92). These studies establish persister heterogeneity within the examined models, but not its prevalence or composition in patient MRD. What remains unresolved is the relative contribution of pre-existing clonal selection, treatment-induced cell-state adaptation, ongoing genetic diversification, and lineage or epigenetic remodeling. Pre-existing DTP-like populations can clearly exist, but therapy can also generate new permissive states through lineage plasticity and stress-adaptive rewiring. Current evidence supports contributions from both selection and induction, with their relative weight likely varying across tumors and treatment contexts. In evidence terms, this conclusion is supported by convergent *in vivo* and residual-disease modeling studies, although prospective patient-level validation remains limited. What still needs strengthening is the prospective linkage of these states to patient-level MRD kinetics and eventual relapse trajectories.

### 10.2 It is also strong that mitochondrial survival circuitry matters, but the exact dependency is probably context-specific rather than universal

The broader mitochondrial-survival model is supported by multiple convergent studies, but the dominant dependency is likely to remain context-specific rather than universal. In 2018, Song and colleagues showed that short-term EGFR inhibition increases MCL-1 synthesis in surviving EGFR-mutant cells and that BH3-mimetic cotargeting could eliminate these early survivors and shrink tumors *in vivo* (93). More recently, Ma and colleagues reported that MCL-1 is elevated in primary resistant and senescence-like drug-tolerant populations, and that combining osimertinib with MCL-1 inhibitors enhanced mitochondrial cytochrome c/Smac release and delayed acquired resistance *in vivo* (94). These studies justify a strong conclusion that apoptotic buffering is central to early persistence. What remains uncertain is whether these states converge on a shared antiapoptotic dependency. Some models appear more MCL-1–dominant, while others point toward BCL-XL, YAP-linked survival, mitophagy, or ferroptosis buffering. That variability is not a nuisance detail; it may be the key reason why seemingly rational combinations perform unevenly across models. The strongest conclusion is not that all residual disease is mitochondria-dependent, but that mitochondrial maintenance and apoptotic buffering can represent recurrent survival solutions in specific EGFR-mutant persistence models. The term “mitochondrial niche” should therefore be used as an organizing hypothesis rather than as a claim of universal dependency. The future task is to define which apoptotic and redox liabilities are shared, and which are lineage-, state-, or context-restricted.

### 10.3 A major unresolved question is whether the external niche is primarily pre-existing, therapy-remodeled, or both

Current evidence supports both pre-existing and therapy-remodeled niche components, although their relative contribution remains unclear. In a 2025 digital spatial profiling study of 25 EGFR-mutant NSCLC samples obtained before and after EGFR-TKI treatment, osimertinib remodeled the tumor microenvironment by altering angiogenic pathways and immune-cell proportions, with fewer plasma cells and a tendency toward greater macrophage infiltration after treatment; notably, the rare tumors that shifted from a lymphocyte-depleted C4 state to an IFN-γ-dominant C2 state subsequently derived durable benefit from atezolizumab/bevacizumab/carboplatin/paclitaxel (95). Yet a separate 2025 spatial whole-transcriptomic study of treatment-naïve EGFR-mutated LUAD showed that PD-L1-high tumors already possessed a more immunosuppressive and exhausted microenvironment, enriched for Tregs, MDSCs, EMT-related programs, and checkpoint molecules, whereas PD-L1-low tumors showed stronger mitochondrial-function programs (96). These studies can be integrated into a two-axis model comprising baseline niche state and therapy-induced remodeling magnitude. The niche is neither fully pre-existing nor simply created by therapy: pretreatment spatial state may define the starting ecology, whereas on-treatment remodeling may determine whether residual disease remains immune-excluded, becomes myeloid-dominant, or shifts toward an IFNγ-enriched state. Serial spatial profiling through the MRD window is needed to determine whether these states are coupled to mitochondrial dependence. Instead, EGFR-mutant tumors appear to enter treatment with unequal baseline ecologies, after which EGFR-TKIs further remodel those ecologies in ways that can either reinforce immune inefficiency or occasionally make later therapies more effective. This is now Level 1–2 translational evidence, because it comes from patient tissues and spatial assays. What is still missing is serial spatial profiling through the true MRD window, rather than only pretreatment-versus-relapse snapshots.

### 10.4 TAK1 is an attractive convergence node, but in this review it should still be treated as a working hypothesis

TAK1 enters this framework because it could, in principle, connect stress inflammation, stromal signaling, and cell survival. In lung cancer, Shin and colleagues showed in 2025 that PYCR1 functionally couples EGFR and TLR signaling, promoting AKT, TAK1, and NF-κB activation; loss of PYCR1 weakened both EGFR- and TLR-induced signaling and suppressed tumor spheroid growth (97). This is highly relevant, because it provides a plausible route by which therapy-shaped inflammatory inputs could converge on a survival-promoting kinase hub. However, it does not yet prove that TAK1 is the central organizer of EGFR-TKI residual disease. The best evidence for TAK1 as a stromal immune regulator currently comes from outside lung cancer. In pancreatic ductal adenocarcinoma, Sheng and colleagues showed that TAK1-high CAFs support an inflammatory, immunosuppressive phenotype; TAK1 loss converted CAFs away from that state, reduced protumorigenic signaling, and increased intratumoral CD4^+ and CD8^+ T cells *in vivo* (98). That cross-tumor result is mechanistically provocative but still extrapolative in the present context. TAK1 is therefore a candidate convergence node rather than an established driver of the mitochondrial niche. Decisive evidence would require TAK1 activation in EGFR-mutant on-treatment residual specimens, compartment-resolved localization to tumor cells or CAFs, and functional suppression of residual survival after TAK1 perturbation in osimertinib-treated EGFR-mutant models, with simultaneous measures of stromal state, mitochondrial stress, and immune competence. Until then, TAK1 should remain a cross-tumor hypothesis rather than a disease-specific mediator of the EGFR-mutant residual niche (99, 100).

### 10.5 Alternative interpretations and boundaries of the mitochondrial-niche model

The mitochondrial-niche model is intended as a testable organizing hypothesis rather than a universal mechanism of EGFR-TKI persistence. Most mechanistic evidence remains preclinical, and direct evidence that ctDNA-defined MRD corresponds to an integrated mitochondria-dependent persister ecosystem in patients is currently lacking. No study has yet simultaneously demonstrated tumor-cell mitochondrial dependence, CAF- or matrix-mediated support, immune dysfunction, and longitudinal residual disease within the same EGFR-mutant system (26, 69). ctDNA positivity identifies residual disease burden, but not its cellular composition, spatial organization, or metabolic state. Parallel or competing explanations include selection of pre-existing resistant clones, ongoing genetic and copy-number evolution, chromosomal instability, lineage plasticity, epigenetic or chromatin-enabled adaptation, bypass signaling, and heterogeneous EGFR expression or drug exposure. The model should therefore be judged by whether it identifies mitochondria-dependent residual states, necessary stromal or immune components, and clinically actionable intervention windows, rather than by whether it explains every form of EGFR-mutant residual disease (14, 31). Clinical translation introduces additional limitations. Most proposed biomarkers lack harmonized assays, validated cutoffs, and demonstrated utility for treatment selection. Residual states are likely to be spatially and temporally heterogeneous, making single-biopsy classification unreliable in some patients (101, 102). Candidate metabolic, stromal, and immune interventions may also have narrow therapeutic windows because their targets perform essential functions in normal tissues. Clinical adoption will therefore require biomarker-enriched prospective trials incorporating target-engagement measures, serial biospecimens, prespecified toxicity monitoring, and evidence that biomarker-guided intervention improves outcome over standard care.

## 11 Conclusion

Therapeutic failure in EGFR-mutant NSCLC has traditionally been interpreted through acquired resistance, including secondary mutations, bypass signaling, lineage switching, and re-expansion of drug-refractory clones. The acquired-resistance framework and residual-state models address different temporal layers of treatment failure and should be considered complementary rather than mutually exclusive. The central argument of this Review is that recurrence should be examined as a branching temporal process in which residual cell states may interact with—or evolve independently of—genetic and lineage-associated resistance mechanisms. Across separate studies, mitochondrial, stromal, and immune adaptations have each been associated with residual survival, but their coexistence and causal integration remain unproven. The mitochondrial-niche framework is therefore best viewed as a context-dependent hypothesis for biomarker-defined residual states rather than a universal model of EGFR-mutant NSCLC. Framed in this way, minimal residual disease should be regarded not only as a low-burden clinical state, but also as a context in which biologically distinct residual cell states may persist. This perspective carries both conceptual and translational implications. Conceptually, it shifts the field away from a static catalogue of resistance mechanisms and toward a temporal model in which drug-tolerant persistence, MRD maintenance, and stable acquired resistance are related but non-equivalent states. The value of this framework will depend on longitudinal studies that determine which residual states are mitochondria-dependent, whether specific stromal or immune components are necessary, and whether their interception improves clinical outcomes with acceptable toxicity. Early trials should prespecify what constitutes residual-disease control, including sustained driver-ctDNA negativity, delayed molecular recurrence, absence of emergent resistance clones, resolution of residual lesions, and pharmacodynamic depletion of defined residual-cell or niche markers. These endpoints remain mechanistic until validated against progression-free survival, overall survival, toxicity, and patient-reported outcomes. More aggressive niche disruption may also impose evolutionary pressure and accelerate resistant-clone selection; future trials should therefore measure response depth, clonal diversity, resistance trajectories, safety, and durability of molecular control. Until such evidence is available, the mitochondrial-niche framework should guide experimental design and prospective trial development rather than routine clinical practice.
